# Fetal Growth Restriction and Its Metabolism-Related Long-Term Outcomes—Underlying Mechanisms and Clinical Implications

**DOI:** 10.3390/nu17030555

**Published:** 2025-01-31

**Authors:** Anca Adam-Raileanu, Ingrith Miron, Ancuta Lupu, Laura Bozomitu, Maria Oana Sasaran, Ruxandra Russu, Solange Tamara Rosu, Alin Horatiu Nedelcu, Delia Lidia Salaru, Ginel Baciu, Cristina Maria Mihai, Tatiana Chisnoiu, Omer Faruk Beser, Vasile Valeriu Lupu

**Affiliations:** 1 Pediatrics, Faculty of Medicine, “Grigore T. Popa” University of Medicine and Pharmacy, 700115 Iasi, Romania; anca.adam-raileanu@umfiasi.ro (A.A.-R.); lucmir@gmail.com (I.M.); vasile.lupu@umfiasi.ro (V.V.L.); 2 Pediatrics, Faculty of Medicine, “George Emil Palade” University of Medicine, Pharmacy, Science and Technology, 540142 Targu Mures, Romania; oanam93@yahoo.com; 3 Faculty of Medicine, “Grigore T. Popa” University of Medicine and Pharmacy, 700115 Iasi, Romania; ruxandra_rss@yahoo.com (R.R.); rosusolange@yahoo.com (S.T.R.); alin_nedelcu@yahoo.com (A.H.N.); delia.salaru@umfiasi.ro (D.L.S.); 4 Faculty of Medicine and Pharmacy, “Dunarea de Jos” University of Galati, 800008 Galati, Romania; ginelbaciu@yahoo.com; 5 Pediatrics, Faculty of Medicine, “Ovidius” University, 900470 Constanta, Romania; cristina2603@yahoo.com (C.M.M.); tatiana_ceafcu@yahoo.com (T.C.); 6 Department of Pediatric Gastroenterology, Hepatology & Nutrition, Cerrahpasa Medical Faculty, Istanbul University Cerrahpasa, 34776 Istanbul, Turkey; ofbeser@gmail.com

**Keywords:** fetal growth restriction, intrauterine growth restriction, small for gestational age, metabolic outcome, thrifty genotype, fetal origin of adult disease, metabolic syndrome, nutrition

## Abstract

The developmental origins of adult disease theory support the concept that undernourished fetuses are at risk of developing metabolic syndrome due to the energy-saving ‘Thrifty Phenotype’. This metabolic plasticity represents an evolutionary adaptation that allows individuals to resist the intense pressure caused by cyclically recurring periods of nutritional deprivation. A comprehensive review was conducted following an extensive literature search in the PubMed/Medline and EMBASE databases concerning reports on fetal/intrauterine growth restriction and its metabolic-related long-term outcomes. We only included articles written in English that were published before 1 July 2024. There are several underlying mechanisms and metabolic and endocrine adjustments shaped by the perinatal environment, and they all contribute to progression towards adult disease. From in utero malnutrition or other insults during the fetal period to fetal programing and postnatal catch-up growth, it is difficult to identify the exact moment when this adaptative phenomenon meant to assure fetal survival and to set children on their own physiological growth curves lose its beneficial effect, establishing the trajectory to obesity, insulin resistance, and other hallmarks of metabolic syndrome. With clinical correspondence to an altered body mass, composition, and eating behaviors, it is evident that the metabolic complications linked to FGR are intricate and arise from disturbances in several pathways and organs, but the underlying processes responsible for the long-term consequences are just starting to be understood. The lack of continuity in perinatal-to-pediatric FGR research sets the challenge of exploring new directions in future scientific opportunities. These will hopefully represent a cornerstone in the management of FGR-related metabolic disorders in children, preventing these disorders from evolving into adult disease.

## 1. Introduction

Despite advancements in both obstetric and neonatal care, fetal growth restriction (FGR), previously known as intrauterine growth restriction (IUGR), remains a challenging issue for medical practitioners as some of the growth difficulties might persist in infancy, childhood, and adulthood. Described as an abnormal fetal growing pattern, FGR characterizes the situation when the fetus is not capable of fulfilling its genetically determined growth potential [1].

FGR affects 8–10% of pregnancies worldwide, with its diagnosis being established before birth [2]. It is characterized by a significant difference between the biometric parameters of the fetus, measured by ultrasound, and the values corresponding to its gestational age [3,4,5]. In the absence of a clinical gold-standard definition, the current literature frequently associates FGR with another medical term, small for gestational age (SGA), commonly defined as a birth weight less than the 10th percentile for gestational age. Low birth weight is a term that conceptually brings together premature, SGA newborns with those affected by FGR. Despite the fact that FGR and SGA are not synonymous, SGA is a valuable variable used for epidemiological study due to the availability of birth weight and gestational age data in vital statistics and other health registries [6].

Normal fetal growth is defined by genetic prospects for growth, but it is influenced by several maternal, fetal, and/or placental variables [7]. Even though many newborns meet the requirements for both FGR and SGA, it is mandatory to distinguish between the two of them. The first implies the existence of an unfavorable intrauterine environment that requires the fetus to re-adapt its metabolic and endocrine systems in order to save energy needed for survival, which may also have an impact on the fetus’s subsequent growth and development [8]. However, the latter term of SGA is not always the consequence of an unfavorable intrauterine environment. SGA can also define a constitutionally small infant whose growth is not affected by any pathological mechanism but is assessed according to several factors including ethnicity, sex, and parental height [9,10,11]. Nevertheless, another distinct feature between the two categories is that SGA newborns might be tiny without carrying a higher probability of a poor perinatal and long-term outcome, whereas FGR may have an estimated weight above the 10th percentile and yet present a higher risk of perinatal morbidity and lifelong negative consequences [8,12].

In regular practice, a normal fetal intrauterine evolution is determined by hereditary background in association with an adequate placental blood supply that is going to ensure sufficient oxygen and nutrients for the fetus to grow. Nevertheless, we must take into account the effect of the hormonal maternal and fetal milieu and the contribution of several growth factors on the development of both the fetus and the placenta, as perturbations in any of these factors, depending on their intensity, length, and time of action, will result in fetal distress or may even lead to the end of the pregnancy [13,14]. As already mentioned, there are many factors that can interfere with fetal growth. Depending on their nature, these factors can be placental, maternal, or even fetal-related, and in most cases, these factors overlap [15]. Despite a large amount of research addressing various facets of FGR in human pregnancies, the underlying cellular and molecular mechanisms underpinning this condition remain intricate and poorly understood.

As is already widely recognized, FGR newborns are associated with increased perinatal mortality and morbidity, being predisposed to long-term health problems because of the unfavorable uterine conditions and/or genetic factors that caused growth restriction [16]. As a consequence, these individuals might face metabolic disorders, cardiovascular disease, and kidney and lung illnesses, as well as neuro-cognitive disorders [17]. Adult morbidities resulting from FGR are complex and include several organs and pathways. In general, terms such as metabolic and nonmetabolic may be used in order to categorize most of the FGR-related morbidities. However, the degree to which these FGR-related effects extends varies between individuals. Metabolic syndrome is not an adult-specific disorder; it is a constellation of metabolic dysregulations that can be identified from childhood to adulthood. Among its central elements are abnormal values for the abdominal circumference and body mass index (BMI), triglycerides, cholesterol, blood pressure, and blood glucose [18,19].

The current narrative review article intended to shed light on the causes and consequences of FGR, with a particular focus on the processes that may link FGR to poor metabolic outcomes during childhood and adult age. We also aimed to raise awareness on the lack of continuity on perinatal-to-pediatric FGR research, setting new directions for future scientific opportunities.

## 2. Materials and Method

A comprehensive review was conducted following an extensive literature search using the PubMed/Medline and EMBASE databases for reports on fetal/intrauterine growth restriction and its metabolic-related long-term outcomes. The search strategy was as follows: [Fetal growth restriction OR Fetal Growth OR Intrauterine Growth OR IUGR OR Fetal growth Retardation OR Fetal growth restriction] AND [Metabolic syndrome OR Metabolic outcome OR Metabolic distress]. To broaden the search parameters and guarantee that all relevant papers were located, the “cited by” feature in Web of Science and Google Scholar was also used. We only included articles written in English that were published before 1 July 2024. Both original and review articles were taken into account. We excluded articles with unclear titles or abstracts that did not reflect the exact purpose of the article.

## 3. Hypothesis on Fetal Growth Restriction and Developmental Origins of Adult Disease

FGR represents an important etiopathological factor in the development of metabolic and cardiovascular diseases in adults. The effects of FGR appear to be most important when they occur in an “unsuitable” postnatal environment where nutritional restriction in utero is followed by postnatal overnutrition, associated with an increased risk of childhood obesity [20]. Both obesity and metabolic syndrome are epigenetically regulated, starting with in utero nutrition and continuing with the postnatal feeding of infants and young children [21,22]. In other words, FGR serves as a valuable model for analyzing the theory of developmental origins.

**Figure 1:**
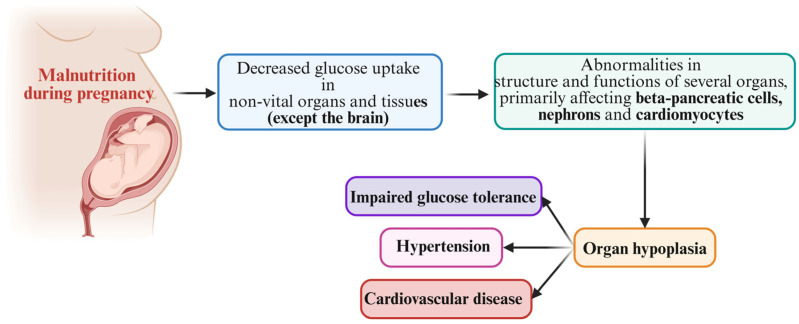
The “thrifty phenotype” hypothesis and the risk of metabolic disease.

It started back in 1962, when the geneticist James Neel first proposed the notion of a “thrifty gene” that conferred a metabolic benefit during famine but encouraged metabolic illness during food abundance, in his attempt to explain the increase in obesity that was behind a rise in diabetes. Accordingly, the argument was based on the idea that, in our early evolutionary history, the existence of some genes that promoted efficient fat deposition would have been desirable because they allowed survival during famines. However, in current times, when food availability is not a real issue, such genes are harmful as they encourage fat deposition in anticipation of a never-ending famine, favoring instead the development of obesity and diabetes [23,24]. Almost 30 years later, in the 1990s, the risks associated with a low birth weight took on a new dimension when Baker’s work was published, suggesting an inverse association between birth weight and the risk of type 2 diabetes and cardiovascular disease in adulthood [25]. His research validated the concept of “fetal origin of adult disease”. Furthermore, to deepen the existing knowledge, he used the “thrifty phenotype hypothesis”, summarized in Figure 1, as an explanation for the correlation observed [26].

The thrifty phenotype hypothesis states that during the critical window of development, a fetus exposed to an unfavorable intrauterine environment reconfigures its endocrine–metabolic behavior in order to adapt to these adverse conditions [26,27]. In other words, for the purpose of conserving energy for survival, the fetus will be giving less nutrition and energy to organs that are not necessary for living, in a brain-sparing effort. As an example of peripheral organs that must adapt to a restricted protein and glucose intake, the muscles and the liver will develop a self-preserving mechanism that will assure their endurance, but with long-term detrimental effects, including a certain degree of insulin resistance [28,29]. Hepatic adaptation increases the risk of dyslipidemia, vascular remodeling increases the odds of endothelial damage and future hypertension, and, altogether, insulin resistance increases the risk of metabolic syndrome progression. This phenomenon is known as ‘fetal programming’, when an insult in utero leads to functional changes in key organs, changes that will persist in postnatal life and lead to a greater risk of later diseases in adult age [30]. A particularly important aspect of this discussion is the “developmental plasticity” of the fetus, which is described as the capacity of a single genotype to create several alternative forms of structure, physiological state, or behavior, in response to specific environmental factors [31,32].

As evidenced by the current literature, the human genome possesses several plasticity mechanisms used to control the expression of genes and proteins [33]. DNA methylation, noncoding RNAs, DNA variations, chromatin, and histone modifications are some of the processes underlying genome plasticity, and they can all be influenced by different factors [34]. Depending on external environmental factors, as well as various internal physiological elements, in an interaction between genes and the environment, these alterations in gene activation and repression will enable organisms to generate several phenotypes from the same genotype [35].

In other words, the variability of organismal phenotypes is frequently facilitated by the variability of gene expression. Alterations in gene activation and repression allow organisms to generate several phenotypes from the same genotype, depending on external environmental factors, as well as various internal physiological elements, in an interaction between genes and the environment [31].

With regard to insulin resistance, a central element in FGR-related metabolic syndrome, a number of potential genes have now been identified as risk factors for its development. In a study on young adults affected by FGR, Vu-Hong et al. [36] demonstrated the existence of a correlation between the insulin gene variable number of the tandem repeat locus and severe forms of FGR [36]. Furthermore, recent evidence points to epigenetic modifications in the pancreatic gene silencing of the Pdx 1 homeobox gene as an example of one such developmental process brought on by growth retardation in utero. In a rat model of intrauterine growth retardation, Park et al. found similar epigenetic adaptations associated with the differential binding of dinucleotide methyl transferase 1 and 3a, as well as modifications in histone acetylation and methylation in the promoter region of the Pdx 1 homeobox gene [37]. Significantly, this epigenetic modification was maintained from two weeks to four months of age and could be responsible for the reduced mass of pancreatic βcells linked to a prediabetic condition in these rats [38].

It is proposed that all of these changes occur via epigenetic alterations to gene expression, independent of modifications to genes’ amino acid sequences. Additionally, it is thought that these epigenetic adjustments that increase the risk of obesity and cardiovascular disease also cause appetite dysregulation, which encourages eating and adipogenesis and promotes progression towards obesity. According to a concept known as “transgenerational transfer of environmental information”, epigenetic variations could be transmitted to future generations, even in the absence of exposure to a harmful intrauterine environment [7,39,40].

As another example of the thrifty genotype’s current existence, we must make reference to Polynesians, known for their increased BMIs, and mention Samoans. Because of their high obesity incidence, Samoans represent a special group that is ideal for the study of novel genetic causes of obesity. In numerous research papers, it has been reported that Samoans display a capacity for fast weight gain that only happens in other populations under forced eating circumstances [41,42]. In the modern obesogenic environment, genetic susceptibility to obesity may have arisen from genetic drift brought on by founder effects, small population sizes, and population bottlenecks or from purported selective advantages from efficient energy metabolism gained over 3000 years of Polynesian island discoveries, settlement, and population dynamics, a dynamic genetic that was transmitted from ancestors [42].

After conducting a genome-wide association analysis of BMI in Samoans, Minster and colleagues found and confirmed a strong association with a missense variant in CREBRF that, when compared to the wild-type protein, increased fat storage and reduced energy use, thereby promoting cellular energy conservation [43]. Further evidence for the possible significance of this polymorphism in organismal energy balance came from the “lean” appearance of flies and mice that lacked the gene’s ortholog [44,45]. In addition to Minster and colleagues, Fu et al. reported that in Samoans, females with the minor A allele of rs373863828 had a higher rate of change in their BMI. Females with the AA genotype had an average BMI growth of 0.30 kg/m^2^/year, which was higher than that of females with the GG genotype [46]. However, in Samoan newborns, the same variant was linked to higher lean and bone mass rather than higher BMI or obesity [47]. Together with evidence of positive selection, these findings support the thrifty variation theory of human obesity and highlight the need to study distinct populations in order to uncover novel genetic contributions to complex phenotypes.

To conclude, the Developmental Origins of Adult Disease Hypothesis, based on the “the thrifty phenotype” hypothesis, explains the fact that a fetus’s capacity to adapt to unfavorable in utero circumstances through epigenetic modifications that take place during the crucial window of developmental plasticity might be an essential contributor to the genesis of adult illness [31].

## 4. Mismatch Concept

To summarize, the notion of the ‘thrifty phenotype’ has played a pivotal role in emphasizing that adverse effects on metabolic health arise from the fetal adaptive response to a suboptimal nutritional environment and the subsequent nutritional mismatch between the resulting phenotype and the postnatal nutritional environment [48].

Therefore, in reaction to stressors, the growing fetus may adjust its size, as well as the structure and function of its tissues in order to maintain survival in utero. However, these changes make the newborn more likely to acquire weight faster when placed in a more favorable postnatal environment. According to Bateson and colleagues [49], if the postnatal environment is similar to the prenatal environment, individuals whose prenatal conditions predicted poor adult nutrition may have better outcomes; on the other hand, if there is an ample supply of nutrients, in contrast with in utero adaptations, notwithstanding their short-term usefulness, the individual’s state of health may well worsen, as an environmental mismatch [49].

## 5. The Catch-Up Growth Hypothesis

Subsequently, in appreciating the long-term metabolic risk in FGR infants, we must take into account the growth pattern that they display in their extrauterine life. The catch-up growth hypothesis supports the idea that fetal malnutrition during pregnancy leads to a low birth weight. As a result, in order to overcome this growth deficit registered at birth, during their first years of life, these children experience catch-up growth, a dynamic process characterized by a growth rate higher than the average for their chronological age and sex [50].

FGR-affected infants usually experience catch-up growth from 6 months to 2 years of age, with approximately 85% of them reaching the appropriate growth for their age and sex at the age of 2 [51,52,53]. There is proof that catch-up growth exerts positive effects on body weight and composition without any alterations to the metabolic profile at one year of age as a compensatory and physiological process. Furthermore, during the first year of life, this accelerated growing pattern is not associated with a reduced insulin sensibility or altered leptin levels. As a matter of fact, Beltrand et al. [54] observed that at the end of the first year of life, when the normal body weight was restored, the growth velocity of infants known to have had FGR returned to normal levels [54].

Offering a different perspective, Hediger and colleagues [55] reported that throughout early life, SGA infants continued to be noticeably lighter and shorter, and they did not appear to catch up between the ages of 36 and 83 months. With reduced muscularity, these children presented shifts in their fat/lean mass ratio, negatively impacting metabolism control [55]. With similar results for body size, but not taking into account body composition, Chakraborty et al. [56] concluded that growth restriction patients did not catch up in terms of weight and height with normal children by the time that they were nine years old and stated that there was no proof linking prenatal growth restriction with obesity in later life [56].

**Figure 2:**
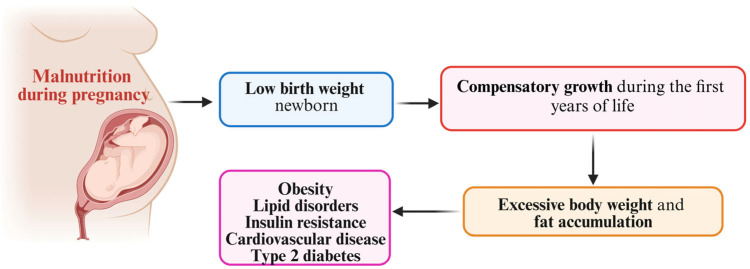
The “catch-up” hypothesis and the risk of metabolic disease.

Nevertheless, several epidemiological studies in the literature are putting forward the hypothesis that individuals with a low birth weight associated with subsequent catch-up growth display an increased risk of type 2 diabetes, obesity, and cardiovascular disease [57,58,59,60]. Although FGR has been correlated with positive effects on a short-term basis, various reports from Europe, Africa, and Asia have come to the conclusion that children with poor weight are 2–8 times more likely to become overweight and/or to have metabolic and cardiovascular diseases [61,62,63,64]. A Finnish study reported that men with a low birth weight and poor weight gain over the course of their first 2 years of life who managed to catch up weight, becoming overweight during childhood, had a 5-times greater mortality rate, in contrast with men who displayed a high birth weight but became lean during childhood [60]. Another Finnish study that recorded changes in body size in 8760 individuals from Helsinki over a period of 11 years reported that coronary events were more likely to appear in individuals with a low birth weight who remained lean during infancy but caught up weight afterwards. In addition, the researchers commented that the growth pattern seen during childhood had a stronger influence on later cardiovascular risk and insulin resistance than BMI measured at a specific age [25]. As a matter of fact, some authors postulate that catch-up growth might represent a state of insulin resistance. Food deprivation or other insults during the fetal period, especially during crucial periods of growth, might result in long-lasting changes to the morphology and functionality of several organs, including the liver, kidney, muscle, adipocytes, and pancreatic beta cells, also linked with a primary neuroendocrine system reset, as described in Figure 2 [65].

With contradictory results across the literature, it is still unclear whether the pattern of prenatal development has a major role in conditioning early catch-up growth or whether the postnatal nutritional environment plays a major role. In other words, is there a contradiction between postnatal nutrition and this early acceleration of postnatal development, or this is a compensatory phenomenon meant to set children on their own physiological growth curves?

## 6. Catch-Up Fat Phenotype and Body Composition in Fetal Growth Restriction-Affected Individuals

An increasing amount of research indicates that newborns with growth restriction have a different body composition than those with normal intrauterine development. A recent systematic review and meta-analysis examined the variations in body composition parameters between newborns with FGR and infants with normal intrauterine growth from birth into their first postnatal months. The authors’ main conclusion was that during their early months of life, infants affected by FGR and SGA were thinner and shorter than those with normal development, with reduced levels of FM (fat mass) and FFM (free fat mass) [66,67,68]. It is a known fact that these children share a different body composition from birth, but in order to understand the metabolic risks that they are exposed to when reaching adult age, we first need to understand their body composition evolution through childhood.

Therefore, we must explore the existence of a new phenotype catch-up fat phenotype, defined by impaired glucose metabolism and hyperinsulinemia. It characterizes children born with a low birth weight who, during their catch-up growth period, gain more adipose tissue than lean mass. In fact, it was reported that among children with a low birth weight, who had a rapid weight catch-up immediately in the postnatal period, extra fat mass and impaired insulin sensitivity were occurrences that could be observed as early as one year of age [69,70,71]. In agreement with this, studies by Ong et al. [58] and Soto et al. [72] came to the conclusion that a weight gain of more than 0.67 DS during the first year of life in SGA infants led to an increased risk of obesity and higher fasting insulin levels [58,72].

Similar, several studies from Spain, Greece and Switzerland reported that from children aged 2 to adolescents who were known to have had a low birth weight, a reduced lean body mass was seen, but no reduction in fat mass, leading to a higher percent body fat compared to children of the same age and weight [55,73,74,75,76]. Furthermore, regardless of BMI, most infants with FGR experience quick catch-up growth throughout infancy [51], followed by higher levels of abdominal fat and enhanced centralized fat distribution in childhood and adulthood [77,78]. Similarly to other research papers, the authors share the hypothesis that the quality of the intrauterine environment and the nutritional status of the fetus, as indicated by its birth weight, may have an adverse influence on the programming of body composition and abdominal obesity in later life, undoubtedly contributing to insulin resistance development and, subsequently, raising the odds of developing metabolism-related disorders [60,77,79,80,81].

In this particular situation in an FGR-affected individual, catch-up growth appears to be a natural endogenous mechanism aimed at restoring body size and composition without adversely affecting insulin metabolism. It is difficult to pinpoint the precise moment at which this physiological process, which attempts to return the body to its normal parameters, becomes harmful, but it clearly plays an important role in the appearance of the metabolic abnormalities known to define metabolic syndrome in adult age.

## 7. Fetal Growth Restriction and Brain–Gut Axis

The brain–gut axis makes an important contribution to body weight regulation. Eating behavior and food intake have the ability to modulate the secretion of different hormones of the gastrointestinal tract, influencing long-term body weight balance [2]. Ghrelin and leptin are key hormones that regulate appetite, maintain metabolic balance, and regulate the neuroendocrine and metabolic response to hunger, with their serum level being regulated by BMI [82,83].

Ghrelin, known as the “hunger hormone”, is a 28-amino-acid peptide predominantly secreted in the stomach, and it is associated with the control of food intake and energy metabolism on the central and peripheral levels, influencing lipogenesis and insulin sensitivity, possessing anti-inflammatory properties, blocking the renin–angiotensin system, reducing sympathetic activity, affecting blood pressure and heart rate, and ultimately being involved in the development of cardiovascular disease (with low values being associated with an increased risk of cardiovascular disease worldwide) [84,85]. Its production throughout fetal development has been shown to begin at 20 weeks of gestational age and increase during the first years of life. Total ghrelin levels fall by 30–50% during late childhood and pubertal development, pointing to a possible influence of sexual hormones on ghrelin synthesis [83]. In the particular situation of newborns that are SGA and affected by FGR, lower ghrelin cord levels have been measured, compared to AGA newborns. Furthermore, when associated with low total ghrelin cord levels, these infants seem to present a slower rate of growth within their first 12 weeks of life [86], supporting a neuroendocrine and metabolic response to malnutrition [74]. In contrast, their cord levels of deacylated ghrelin (DAG), one of the two circulating forms of ghrelin, responsible for downregulating nutrient intake, was found in a lower amount in SGA than AGA [87]. Similar findings have been made regarding serum DAG levels in the blood of obese individuals [88,89], suggesting a possible contribution by this hormone to the formation of the “thrifty phenotype” as defined by Barker [26,90].

Leptin is a hormone known to be involved in signaling from energy stores to central regulators, appetite regulation, and taste perception. In addition, it is accepted as an adipose tissue marker, with leptin receptors found in the kidneys, lungs, bone, or cartilage, or even the hypothalamus, modulating the growth hormone–IGF axis [91].

Human studies have revealed that whereas ghrelin levels in the blood are reduced in obesity, insulin resistance, and type 2 diabetes, they are elevated in anorexia and cachexia [92,93,94]. In this context, ghrelin interacts with other peptides involved in appetite control, such as adiponectin and leptin, by sending signals to the central nervous system that activate orexigenic neurons and deactivate anorexigenic ones. When fat reserves are sufficient, leptin, produced in the adipose tissue, signals to the brain to reduce food intake [95]. Leptin is a component of human milk, and it is currently accepted that it contributes to the postnatal programming of a healthy adult phenotype, with a major role in obesity prevention [96]. It is also regarded as a pro-inflammatory adipokine, contributing to mild inflammation that is linked to an increase in fat tissue [95]. The physiological role of leptin appears to be unsuccessful in obesity, leading to high levels of serum leptin (known as leptin resistance), which support the ongoing low-grade inflammation that contributes to chronic illness [96]. However, just like leptin, ghrelin’s mode of action is compromised in obese individuals, whose ghrelin levels decrease in response to a persistent rise in leptin serum concentration [92].

There is proof that there are low leptin concentrations in the cord blood of infants with FGR, indicating lower adipose tissue deposition and pointing to leptin involvement in the pathomechanism of FGR [97]. This finding is associated with maternal elevated leptin levels in pregnancies being affected by FGR [98]. With regard to its biological functions during the fetal period, recent research has shown that cord leptin can predict anthropometric results at three years of age, as well as weight gain during the first six months of life [91,97].

Concordantly, cord leptin was found in lower levels in SGA newborns compared to AGA controls, whereas, at the end of the first year of life, it was found in higher levels than would normally be expected [97]. During their first year of life, children born with FGR consistently demonstrate high serum leptin values with respect to BMI. In FGR-affected females, these elevated levels seem to persist across time. Growth-restricted girls exhibit a higher insulinogenic index and higher leptin levels than girls born with an appropriate weight, while having an identical body composition and BMI [33]. High serum leptin concentrations may indicate an adipocyte deficiency as a result of the unique time course of adipose tissue development in fetuses and children born with FGR. These children may acquire an adaptative leptin resistance that is beneficial to their catch-up growth [99]. However, in the long term, these elevated leptin levels might favor fat tissue accumulation, possibly associated with increased insulin resistance and laying the foundation for future metabolic disorders [99,100].

Furthermore, as a result of the FGR programming of leptin secretion, those who have experienced FGR may have abnormal leptin levels and altered feeding preferences both in childhood and adulthood [101]. Besides endocrine and metabolic adjustments, fetal programming might also be responsible for an alteration in human dietary choices, subsequently influencing the development of food preferences. This might be possible through hypothalamic–pituitary–adrenal (HPA) axis regulation, secondary to altering the hypothalamic circuitry, which is known to influence appetite control, and by affecting the mesolimbic pathways, be responsible for the enjoyment generated by consuming exceptionally delicious, favored meals [102]. A similar reaction was proven to be found in Prader–Willi Syndrome (PWS), known to be associated with the gradual development of severe obesity and the early onset of hyperphagia with food-seeking behavior, unless eating is immediately restricted [103]. In addition, regarding anorexigenic hormones, several studies have reported extremely high levels of leptin, maintaining a positive relationship between BMI and circulating levels of leptin [104,105]. Interestingly, PWS patients at all ages have been found to have elevated ghrelin levels, as well, both before to the onset of obesity and following the onset of hyperphagia in older children and adults [106]. Hyperghrelinemia has been proposed as a possible cause of PWS weight gain and increased hunger because of its orexigenic activity [107,108]. However, it is yet unknown exactly what mechanisms underlie ghrelin dysregulation in this disease.

Children affected by FGR have an intensified adrenocortical reaction to stress [109]. Both animal and human studies have shown that glucocorticoids increase palatable eating by stimulating behaviors mediated through “reward” pathways [110,111]. When favored meals are consumed along with high insulin and glucocorticoids levels, abdominal fat tissue accumulates more quickly.

An interesting study related to long-term FGR consequences was reported by Barbieri and colleagues [112], who aimed to evaluate the innate dietary preferences of individuals affected by FGR. Their most important findings indicate that FGR women have a greater intake of carbohydrates, even after taking into consideration different socioeconomic factors. This was followed by a decrease in protein ingestion, resulting in an increased ratio of carbohydrates to protein. Furthermore, the waist/hip ratio was greater among women with FGR than for those not affected by FGR [112].

Numerous studies indicate that, at least in the early stages of life, the thyroid and the hypothalamic–pituitary–adrenal axis may control prenatal and postnatal development in children born with SGA [113,114,115]. Cianfarani and colleagues found that SGA children with attenuated catch-up growth had greater TSH concentrations, indicating that thyroid function may be involved in intrauterine reprogramming, which might then impact postnatal development [116]. Thyroid hormones are also known to control calorie intake and expenditure, which is crucial in maintaining energy balance. As a result, disorders linked with thyroid hormone dysregulations are characterized by changes in body weight and food consumption [117].

Although further research is needed in order to fully establish the significance of these findings, the current literature supports the hypothesis that changes in prenatal nutrition affect ghrelin and leptin sensitivity and may have long-term effects on the regulation of body weight, fat mass, insulin sensitivity, and eating behavior.

## 8. Discussion

To elucidate the connection between FGR and different forms of metabolic dysregulation, it is important to take into account the possible interplay between genetic variables and the adverse effects of the intrauterine environment on fetal growth. The FOAD theory claims that events occurring during early development exert a significant influence on an individual’s susceptibility to developing adult diseases in the future. The complex interaction among an individual’s genetic background, early-life programing, and a subsequent lifestyle marked by inadequate alimentation techniques leads to the emergence of the thrifty ’catch-up fat’ phenotype—a pivotal occurrence in the progression towards obesity and certain disorders that are commonly associated with insulin resistance.

In order to ensure the efficient management of FGR-affected newborns after birth and to reduce the long-term effects of inadequate prenatal development, it is crucial that we investigate the prenatal pathways that may initiate metabolic dysregulations. Although metabolic syndrome represents a popular topic and has caught the attention of many researchers in both adult and pediatric populations, there is a lack of studies focused on particular sub-groups of the population, i.e., FGR-affected children and adolescents. From different biomarkers of FGR in the mother- and fetus-associated microbiome and metabolomic signatures and specific epigenetic mechanisms known to modulate the dynamic placental epigenome to novel technologies used in perinatal FGR prophylaxis and treatment, all recent available medical data are focused on FGR perinatal events.

There is a lack of knowledge regarding the evolution of FGR-affected children beyond their first 1000 days of life. There are scarce data and a relatively small number of clinical studies on this specific topic. The available results do not provide us with a suitable viewpoint on the onset and evolution of these metabolic dysregulations, leaving a knowledge gap regarding FGR-related metabolic syndrome during childhood and adolescence.

Moreover, due to some postnatal protective physiological mechanisms, not all FGR-affected individuals develop metabolic syndrome later in life. From catch-up growth to postnatal overnutrition, when do mechanisms turn from physiological to pathological, and when do the first signs of metabolic distress appear? There is proof of a strong correlation between adult metabolic syndrome and FGR, but it would be helpful to identify metabolic syndrome’s early manifestations in the pediatric population, in order to initiate appropriate management and halt progression towards adult disease.

However, we share the belief that the results of future research could be helpful in generating adequate screening, preventive, and management policies relating to metabolic syndrome in this particular pediatric population. By translating theoretical insights into clinical practice, we anticipate a rise in the quality of life of FGR-affected children that have associated metabolic disorders and an important reduction in the economic burden of metabolic disease-related care in both the pediatric and adult population.

## 9. Conclusions

Due to recent advances in modern technology, there is a better understanding of the mechanisms by which the human phenotype, from infancy to adulthood, can be customized by the perinatal environment and early-life experiences. With clinical correspondence to an altered body mass composition and to altered eating behavior, it is evident that the metabolic complications linked to FGR are intricate and arise from disturbances to several pathways and organs. Current studies cannot provide the complete picture in terms of FGR-affected children’s progression towards metabolic disease. However, the critical influence of FGR in determining long-term health outcomes emphasizes the need for these children to be carefully monitored for the early development of metabolic syndrome, as its elements may manifest during childhood, requiring diligent evaluation and follow-up.

## Data Availability

No new data were generated.
